# Metabolomics Community in Russia: History of Development, Key Participants, and Results

**DOI:** 10.3390/biotech9040020

**Published:** 2020-10-25

**Authors:** Elena E. Balashova, Dmitry L. Maslov, Oxana P. Trifonova

**Affiliations:** Institute of Biomedical Chemistry, Pogodinskaya st.10, 119121 Moscow, Russia; dlmaslov@mail.ru (D.L.M.); oxana.trifonova@gmail.com (O.P.T.)

**Keywords:** metabolomics, Russian science, mass spectrometry

## Abstract

Metabolomics is the latest trend in the “-omics” sciences, of which technologies are widely used today in all life sciences. Metabolomics gave impetus to the description of biochemical processes that occur in many organisms, search for new biomarkers of disease, and laid the foundation for new clinical laboratory diagnostics. The purpose of this review is to show how metabolomics is represented in Russian science, what main research areas were chosen, and to demonstrate the successes and main achievements of Russian scientists in this field. The review is dedicated to the 10th anniversary of Russian metabolomics and also touches on the history of the formation of Russian metabolomics and prospects for the future.

## 1. Introduction

**Figure 1:**
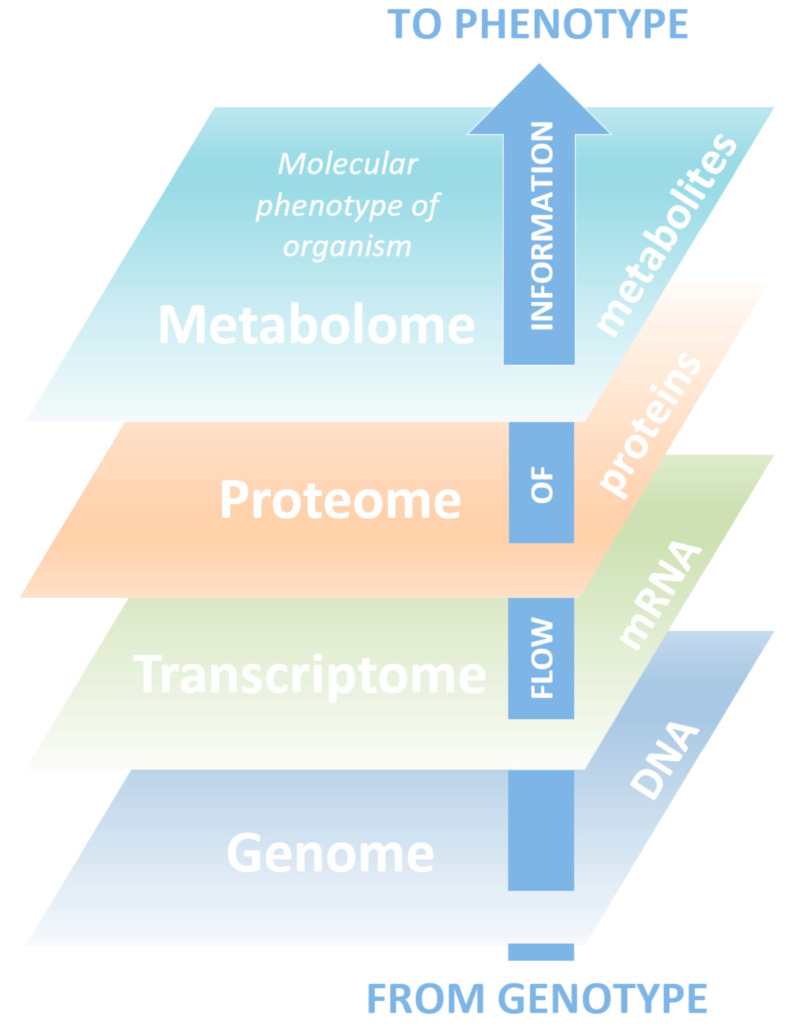
The sequential interconnection of “-omes” in a living system, which reflects the flow of information encoded in genes to the molecular phenotype of an organism—a metabolome.

Metabolomics is known as a science in which technology platforms are aimed at identifying and quantifying low molecular weight compounds (metabolites). Metabolites are substrates and products of almost all biochemical reactions taking place in the body, play a key role in energy generation, signal transmission in the cell, and carry information about the physiological state of a living organism and ongoing pathological processes [1,2,3]. Metabolomics is a relatively new science and the youngest of the triad of basic “-omics”, including genomics and proteomics, which systematically describe biological objects (Figure 1).

The term “metabolome” was first introduced in 1998, but the main development of metabolomics was observed after 2010 [4], largely due to the use of new, constantly improving high-performance analytical methods and comprehensive bioinformatics data processing [5,6]. Along with other “-omics”, metabolomics research focuses on directions where achievements can bring significant benefits to humanity. Generally, metabolomics studies are able to give a representation of the biochemical processes that underlie the body’s reaction to internal and external stimuli.

## 2. Metabolomics in Russia

The first systemic development of “-omics” science in Russia started in the early 2000s at the Institute of Biomedical Chemistry (IBMC) in Moscow. By that time, IBMC, dealing with problems of biological and medical chemistry under the leadership of academician of the Russian Academy of Sciences (RAS) Alexsander I. Archakov, was actively developing post-genomic technologies, such as transcriptomics and proteomics [7,8,9,10,11,12,13]. In 2000, the first in the country Department of Proteomic Research and, in 2003, the “Human proteome” Core Facility was created at IBMC. That allowed to move on to the concept of systems biology, the working definition of which at that time was “the study of biology as an integrated system of genetic, protein, metabolic events that are constantly changing and interdependent” [14]. That is, the main task of Core Facility was to obtain and integrate proteomics, transcriptomics, and metabolic information for a more holistic study of living organisms. The technical base and technological capabilities accumulated at the IBMC became a growth point for such studies and contributed to the opening of the first metabolomics laboratory in the country—“Laboratory of mass spectrometry metabolomic diagnostics” under the supervision of Dr. Petr G. Lokhov. After some time, various metabolomic studies of biological body fluids were performed in the laboratory to characterize biological objects at the molecular level to create metabolites-based diagnostics. The main material of the study was human blood plasma, widely used in the clinic and the most informative in terms of laboratory diagnostics body fluid. The main method for studying plasma metabolites was mass spectrometry analysis. The first metabolomics data obtained in the laboratory was published in 2009–2010 in the journals *Metabolomics* and *Biomeditsinskaya khimiya* [15,16,17].

In recent years, metabolomics has been developing in various fields of Russian science. Scientists study the factors that increase the risk of diseases in humans, the effects of environmental factors, the presence of pathological processes, the patient’s state and the effectiveness of drug therapy. In several research areas metabolomics approaches are used to better understand the mechanisms of pathological processes development and the aging process, in both humans and animals (Table 1).

Significant results were achieved at the International Tomography Center, Siberian Branch of RAS (Novosibirsk), where the Mass Spectrometric Research Center for Collective Use of the Siberian Branch of RAS was established in 2010 under the supervision of Dr. Yuri P. Tsentalovich. At the preliminary stage, the main task of the Research Center was development of modern mass spectrometers. In 2015, on the basis of the Research Center the laboratory of proteomics and metabolomics was opened. Now the laboratory conducts work aimed at studying changes in the biochemical composition of tissues during aging and with the development of ophthalmic diseases, in particular cataract and keratoconus. This work is directed to the developing of new approaches for diagnosis, prevention, and treatment of these diseases (Table 1).

Every year, the relevance of using metabolic technologies has been increased in various fields of science. A large area is devoted to metabolic analysis of plants, and more and more studies performed not on model objects, but using the genetic resources of cultivated plants (wheat, rice, potatoes, peas, corn, etc.) have appeared. Thus, the joint work of the group of scientists from St. Petersburg is devoted to the study of metabolic processes occurring in potato, which is one of the ten most popular agricultural cultures. The group includes scientists from Saint Petersburg State University, Vavilov Research Institute of Plant Industry, and Komarov Botanical Institute (Table 1).

The standardization of metabolomic analysis protocols and methods for the results processing allows the use of metabolomics not only as an essential component of fundamental research, but also as the basis for monitoring collection samples of potato varieties and created hybrids. The obtained data indicate the promise of such approach for phenotyping various potato genotypes, as well as for identifying forms resistant to various types of adverse effects.

## 3. Metabolomic Studies of Human Blood in Russia

Recently, the role of metabolomic studies of human blood in clinically-oriented scientific research has become increasingly important both to search for biomarkers of diseases and to study processes developing in the body, as well as to evaluate the effectiveness and toxicity of drugs [39,40]. Metabolomics make it possible to take into account the influence of all possible factors (both endogenous and exogenous) that affect the body, making conclusions about the mechanism of the disease, diagnostic markers relevance, and predict individual variations in response to drug treatment. Analysis of the blood metabolome, which allows obtaining information on hundreds and thousands of metabolites simultaneously, already shows significant results in solving a large number of scientific and clinical problems. The possibilities of such analysis are described in detail in the review by Lokhov et al. [41]. The greatest success in this field was achieved using direct injection of a low-molecular fraction of blood sample into an electrospray ionization source of a quadrupole time-of-flight mass spectrometer (DIMS, direct infusion mass spectrometry) [42,43]. The resulting mass spectrum reflected the picture of the metabolome that with subsequent statistical analysis in the group with healthy volunteers metabolomes made it possible to establish the normativity of the metabotype, identify xenobiotics, detect low molecular weight biomarkers of diseases, and apply metabolomic diagnostic signatures [41]. It should be noted that for such metabolomic analysis no more than 10 μL of blood is required, therefore, the sample amount is determined exclusively by the convenience of the manipulations and the sampling could be carried out even outside the laboratory, for example, at home using the “dried blood spot” method [44].

In the late 2000s, the group led by academician of the RAS Archakov at IBMC investigated the possibility of using the method of metabolic fingerprinting of blood plasma [45], which allows determining thousands of metabolites in one biological sample, for the diagnosis of the second stage of prostate cancer, one of the most common types of cancer in men [16]. The obtained data on the sensitivity (95%), specificity (96.7%), and accuracy (95.7%) of the diagnosis significantly exceeded the data of the enzyme-linked immunosorbent assay for prostate-specific antigen (PSA test) (35%, 83.3% and 51.7%, respectively) for the same patient samples. The area under the ROC curve (AUC)—one of the most commonly used methods for assessing the diagnostic significance of identified biomarkers [45]—was 0.994, which suggests that the proposed approach to the diagnosis of prostate cancer based on metabolic fingerprinting is effective and applicable in clinical practice [16]. By statistical analysis of the peak intensities of the metabolites in the mass spectra, it was found that six different metabolites are specific for prostate cancer. Two metabolites, acylcarnitine and arachidonoylamine, have an AUC of 0.97 and 0.86, respectively, that is higher than in the PSA test (0.59), which defines these metabolites as potentially suitable markers for early diagnosis of prostate cancer [15].

The first half of the 2010s, the group led by academician of the RAS Ivan I. Dedov at the Endocrinology Research Center (Moscow) initiated a study of the possibility of using metabolomics to diagnose impaired glucose tolerance and use it in clinical practice as an alternative to the existing glucose tolerance test [18]. Impaired glucose tolerance (IGT) is a prediabetic condition that is associated with insulin resistance and an increased risk of cardiovascular disease. Moreover, it has been shown that IGT precedes type 2 diabetes for many years [46]. Currently, the oral glucose tolerance test (GTT) is the “gold standard” for detecting IGT. However, this test showed poor reproducibility, despite being considered useful for the diagnosis of IGT, as well as diabetes and other cardiovascular risk factors [47,48,49]. Additionally, GTT takes a long time (about 2 h), and some people may experience hyperglycemic shock during that time. Therefore, a faster and more reproducible test for the diagnosis of IGT is extremely important task.

**Figure 2:**
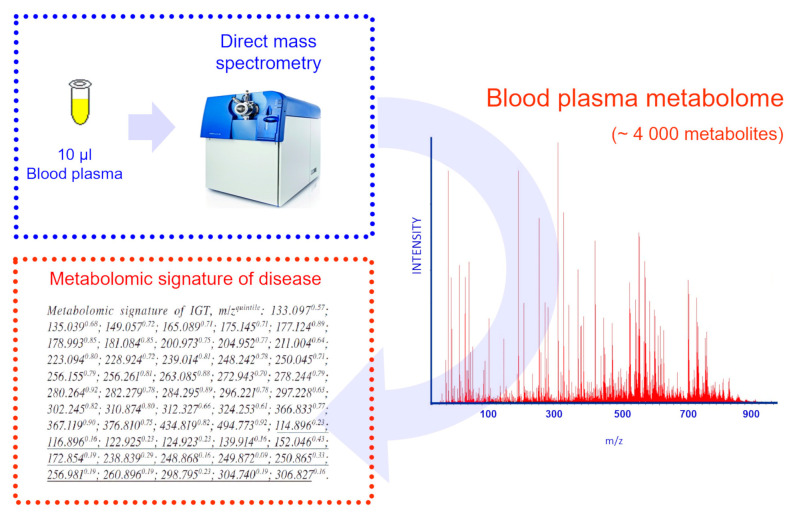
Obtaining a diagnostic signature on the example of impaired glucose tolerance (IGT). Blood plasma samples were treated with methanol to precipitate the protein, and low molecular weight fractions were analyzed by direct injection of blood plasma metabolites into an electrospray mass spectrometer source. Metabolite ions that showed a statistically significant association with IGT were included in the diagnostic signature. For ions included in the signature, correspondence to specific metabolites in the metabolite databases was established. The mass spectrometric signature is written in a unified form: the molecular weights of the ions of substances detected by direct mass spectrometry of blood plasma are indicated, the threshold value in quantiles is indicated in the upper register. If the threshold value is exceeded, then the diagnostic score (mass spectrometry-based glucose tolerance test score) should be increased by one.

The study of IGT was conducted in conjunction with IBMC. Direct mass spectrometry analysis of blood plasma metabolites in this study provided a fast, one-step and reproducible approach for metabolite analysis. Moreover, this method can serve as a prototype for clinical tests that can replace the current glucose tolerance test with a more patient-friendly test. A total of 51 ion metabolites that are closely related to IGT were found [18]. The area under the ROC curve (AUC) was 0.93 (accuracy 90%, specificity 90%, and sensitivity 90%). The corresponding reproducibility was 85%. Identified metabolites corresponding to risk factors previously associated with the development of diabetes [18], were used to compile the metabolomic signature (signature is a set of variable values that forms a specific picture (Figure 2)).

The National Medical Research Center of Oncology (Moscow) together with IBMC have developed a method for the early diagnosis of lung cancer, as one of the most common types of cancer and the main cause of cancer death in Russia [19]. Early detection of lung cancer can significantly reduce mortality. Therefore, it is extremely important to develop laboratory tests to detect human lung cancer, including clinically asymptomatic stages. To this end, mass spectrometric analysis of metabolites was carried out on blood samples taken from patients with lung cancer and age-related controls. The resulting mass spectra were converted to binary format, aligned, reduced to several variables using the cluster analysis (principal component analysis, PCA) and, finally, classified as cancer cases compared to the control using the support vector machine (SVM) method. Repeated testing of random subgroups of samples showed a classification accuracy of 93.3% (sensitivity 94.1%, selectivity 92.4%) that convincingly indicates that DIMS of a low molecular weight blood fraction provides great clinical potential in the diagnosis of early human lung cancer [19].

During the study, hundreds of metabolites associated with lung cancer were detected, and at least 70 metabolite ions were very closely related to the presence of lung cancer [20]. Research has shown that these metabolites potentially could be markers and, therefore, may be associated with an increased risk of developing lung cancer in population studies [20]. Thus, the findings of the study provide a strategy for the prevention and early detection of lung cancer. For example, monitoring these cancer-related metabolites provides a tool, using which doctors can identify people at high risk of developing lung cancer. Accordingly, people at high risk should try to minimize their exposure to risk factors and take advantage of other diagnostic capabilities that already exist in clinical practice. This strategy has the potential to reduce lung cancer and increase patient longevity, contributing to the early detection of cancer.

In the late 2010s, IBMC together with the group led by academician of the RAS Michael V. Ugrumov from the Institute of Development Biology (Moscow) and the group from Kazan State Medical University (Kazan) have tested the new method for the early diagnosis of Parkinson’s disease (PD) based on the metabolomic analysis of blood plasma [21]. Metabolites showing a strong association with PD were included in the diagnostic signature, and the corresponding characteristics for the diagnosis of PD were calculated [50]. The area under the ROC curve (AUC) for the diagnosis of PD was 0.95 (accuracy 94%, specificity 95%, and sensitivity 94%). The metabolites identified in this study were consistent with factors previously associated with the development of PD [21]. Metabolomic studies of PD by Russian scientists are ongoing [51,52].

One of the latest metabolomic studies of IBMC was carried out in collaboration with the Scientific Research Institute of Nutrition (Moscow) [22]. As mentioned above, at present scientists use only a small part of the information contained in the blood metabolome. Identification of metabolites is a significant problem because only well-separated compounds in high concentrations can be easily identified in complex biological samples. However, new approaches have emerged that improve the identification of compounds. The recent development is the identification of compounds based on their participation in certain biological processes [22]. In this work the approach was first applied to identify metabolites in blood samples and used to study the blood plasma of obese patients. It has been found that the proposed approach provides a statistically valid overview of biochemical pathways, thus providing additional molecular-level information on obesity. It has been shown that the progression of obesity is accompanied by noticeable changes in steroidogenesis, androstenedione metabolism, and androgen and estrogen metabolism [22]. It is expected that this algorithm is suitable for studying other metabolic diseases, as well as for monitoring the body’s response to treatment.

**Figure 3:**
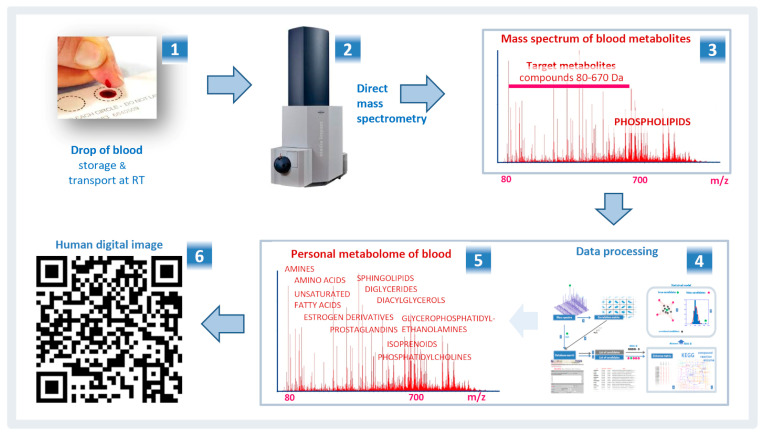
The scheme for obtaining a human digital image based on the mass spectrometry data of a blood metabolome. A dry spot of capillary blood on filter paper is sent to the laboratory for metabolomic investigation (**1**). The low molecular weight substances are extracted from a blood sample and injected directly into the ionization source of the mass spectrometer (**2**). The mass spectrum of blood metabolites (**3**) obtained by DIMS are processed (**4**) following the protocol presented in the publication [25]. The personal blood metabolome (**5**) obtained from the data processing is used to create a human digital image, which is presented as a QR code (**6**). Adapted from [25].

The most recent developments of the Russian scientists in the field of metabolomics include the development of digital technologies. The general trend towards digitalization of technologies in the economy was a prerequisite for the introduction of precision digital methods of laboratory diagnostics in medicine. The Russian scientists have proposed the method for digitizing a human blood metabolome, measured according to the standard protocol, to obtain a digital image of a particular patient [25] (Figure 3). The digital image contains information sufficient to accurately diagnose any disease. It is compact, portable on any digital and paper media, and can be read by ordinary mobile devices (smartphones). The digital image is convenient for cataloging and archiving medical and telemedicine data. In addition, it is also suitable for monitoring the physical condition of a person and conducting longitudinal and population-based medical studies [25]. The widespread use of a human digital image, its further testing on large sets of samples, including cohorts of patients with various diseases and stratified by sex, age, and lifestyle, will help to make this approach more reliable and unified and clarify the limitations of its use in medicine.

## 4. Therapeutic Drug Monitoring

One of the potential uses of metabolomics is the identification of metabolic profiles in body fluids that can predict the effectiveness and toxicity of drugs, the so-called pharmacometabonomics focused on the personalization of drug therapy [53]. The metabolic profile reflects the metabolism of xenobiotics in the body. That is, the metabolic profile contains all information necessary to calculate the effective dose of a drug for a particular patient, taking into account not only the characteristics of the individual pharmacokinetics of the drug, but also the body’s response to it [54,55]. Several works by the Russian scientists demonstrate the success of metabolomics in this direction [56,57].

A new method of therapeutic drug monitoring (TDM) based on direct mass spectrometry of a low-molecular-weight fraction of a sample was developed by scientists at the laboratory of mass spectrometric metabolomic diagnostics of IBMC [56]. This technique allowed to conduct TDM of all drugs involved in the study. The versatility and high performance of direct mass spectrometry greatly simplify its widespread use. Moreover, the possibility of using the method in most cases of drug therapy is considered as a tool for monitoring drug doses, rationality of drug therapy and the quality of drugs used. Additionally, the method can be used as the main means of improving the quality and personalization of drug therapy, providing a prediction of the individual variability of the drug reaction and monitoring the effectiveness of drug treatment, adjusting it, taking into account individual patient parameters [56].

In addition, together with the group led by academician of the RAS Ivan I. Dedov at the Endocrinology Research Center (Moscow) a new method for analyzing human blood lipids (lipidome) was developed based on direct mass spectrometry of a low-molecular-weight lipophilic fraction of blood plasma [57]. The technique allows quantifying hundreds of different types of lipids, and changes the current understanding of the diagnosis of lipid disorders and related diseases. The method has shown its versatility and speed, which greatly simplifies its widespread use. This method is applicable for the diagnosis of atherosclerosis, diabetes, cancer, and other diseases. Detailing the lipid composition of plasma using mass spectrometry makes it possible to evaluate the effectiveness of drug therapy and optimize it for cardiovascular diseases with phospholipid drugs [57].

## 5. Metabolomics of Aging

Today, no one doubts that metabolomics is a promising tool for studying aging, since it is a powerful tool for cataloging changes in the body that occur over time at the molecular level. Aging is a determined process in living organisms, which is characterized by a gradual decrease in physiological activity and reproductive ability, as well as an increase in the frequency of mortality over time. Nevertheless, aging remains one of the most mysterious and not yet fully studied biological phenomena. By measuring numerous small molecules that represent the entire spectrum of metabolic pathways metabolomics can potentially help identify processes that are associated with aging or even lead to it [58,59,60].

Several animals show a different rate of aging, being long-lived and short-lived species, and provide an ideal model to study the mechanisms of aging. The diversity observed in life expectancy among the fish species gives the opportunity to study the mechanisms responsible for significant differences in the rate of aging [61], and long-lived species (slightly aging) can be regarded as a kind of anti-aging model. The study of these species can facilitate the identification of ways to effectively protect against age-related degenerative processes. On the other hand, rapidly aging species can be considered as a model of accelerated aging. Comprehensive studies of various types can help identify mechanisms associated with the rapid development of age-related pathologies [62,63].

In the laboratory of mass spectrometric metabolomic diagnostics of IBMC the first studies of metabolome of fishes with different aging rates were carried out [28,29]. Metabolome profiling of blood plasma from fishes with various aging rates—negligible (*Pike Esox Lucius* and *Sterlet Acipenser ruthenus*), gradual (*Zander Sander lucioperca* and *Perch Perca fluviatilis*), and rapid (*Chum Salmon Oncorhynchus keta* and *Pink Salmon Oncorhynchus gorbuscha*)—were evaluated using direct mass spectrometry. A metabolomic-based study of three well-phenotyped fish cohorts with different aging rates was designed to identify blood metabolites related to the aging rate and assess the relationship of identified metabolites with longevity. Metabolomic analysis revealed 15 metabolites in the classes of dipeptides, fatty acids, glycerolipids, phosphoethanolamines, and phosphatidylcholines, which were largely associated with the rate of aging, regardless of the type of fish [28].

In continuation of the work, metabolic profiling of skeletal muscle of fish with various aging rates was carried out [29], since a progressive decrease in muscle mass and strength, leading to a deterioration of the physiological functions of the body, as well as the development of age-related disorders, is one of the most noticeable signs of aging [64]. Skeletal muscles play a key role in maintaining a healthy and active lifestyle, as they participate in many important functions: controlling movement and posture, physical function, participating in metabolism (for example, skeletal muscles are crucial for maintaining glycemia), etc. Unfortunately, the knowledge of the pathophysiology of loss of muscle mass and strength during aging is still limited [65].

A metabolomic study of skeletal muscles by direct mass spectrometry on a quadrupole time-of-flight mass spectrometer was also carried out on three groups of fishes. The first group included negligibly senescent species of fishes (pike (*Esox Lucius*) and sterlet (*Acipenser ruthenus*)), the second group included gradually senescent species with gradual aging, the same as observed in many species of mammals of the same size (zander (*Sandra lucioperca*) and perch (*Perca fluviatilis*)), and the third group are species with a very short life cycle (chum salmon (*Oncorhynchus keta*) and pink salmon (*Oncorhynchus gorbuscha*)). Multivariate analysis of metabolomic profiles allowed to detect of about 80 features associated with amino acids, lipids, biogenic amines, intermediates of glycolysis, glycogenolysis, and citric acid cycle, which correlate with fish lifespan [29].

## 6. Cataract Pathogenesis

The laboratory of proteomics and metabolomics led by Yuri P. Tsentalovich (International Tomographic Center of the Siberian Branch of the RAS, Novosibirsk) is focused on one of the most interesting directions in Russian metabolomics. The laboratory studies the biochemical and photochemical processes that occur in living organisms and are responsible for the development of diseases [30,31,32].

One of the areas of laboratory research is the study of the metabolomic composition of human and laboratory animals (rat, rabbit, calf, fish) tissues, since the development of pathological processes leads to significant changes in the metabolomic composition of the tissue: a decrease or increase in the concentration of various metabolites. Changes in the metabolomic content of tissue are studied by chromatography, mass spectrometry, and nuclear magnetic resonance (NMR) spectroscopy. The main attention is focused on tissues and biological fluids associated with the eye: the lens, cornea, vitreous humor, and blood. The main goal of the work is to determine changes in the biochemical composition of tissues during normal aging and the development of eye diseases, such as cataracts and keratoconus. Using this information, one can understand the mechanisms of the formation and development of these undesirable processes and evaluate the effectiveness of drugs for the prevention and treatment of these diseases.

In 2016, a combination of NMR spectroscopy and HPLC-MS methods established a quantitative metabolomic composition of biological tissues and human fluids obtained from donors during life and after death. Identification and establishment of concentrations of a wide range of metabolites in the samples of the cornea, lens, serum, and intraocular fluid were carried out. The results can be used to diagnose ophthalmic diseases, as well as to better understand the molecular mechanisms of disease development [30].

In 2017, changes in the metabolomic composition of the human lens due to the development of cataracts were established, which later made it possible to establish detailed mechanisms of cataractogenesis and propose new approaches for the prevention and treatment of age-related cataracts [31]. Concentrations of 86 metabolites were determined for four groups of samples, including the lens of the eye and intraocular fluid from cataract patients and human corpses. It was shown that in the cataract lens the most common metabolites are (in decreasing order): myo-inositol, lactate, acetate, glutamate, and glutathione, and in the intraocular fluid: lactate, glucose, glutamine, alanine, and valine. The concentration of most metabolites in normal post-mortem samples of the lens and intraocular fluid is higher than in samples of patients with cataract [32].

The comparison of the concentration of metabolites in the lens and the respective intraocular fluids shows that the metabolites most important for lens protection are synthesized in the epithelial cells of the lens. Low levels of antioxidants, UV filters, and osmolytes were found in cataract lenses that cannot be explained by post-mortem changes in the normal lens; this indicates that age-related cataract development may occur due to lens epithelial cell dysfunction.

## 7. The Metabolomic Basis of the Host’s Response to Helminth Infection

A joint project of the Laboratory of Clinical Metabolomics of Tomsk State University and Central Research Laboratory of the Siberian State Medical University (Tomsk) is devoted to a comprehensive description of the metabolomic response to opisthorchiasis (more precisely, *Opisthorchis felineus*). Infection caused by trematodes of the Opisthorchiidae family triggers the development of pathologies of the hepatobiliary system, such as chronic forms of cholecystitis, cholangitis, pancreatitis, and gallstone disease, and increases the risk of intrahepatic cholangiocarcinoma.

The work was done on an animal model. Thirty golden hamsters were divided into three groups: with severe infection (50 metacercariae per hamster), with mild infection (15 metacercariae per hamster) and uninfected. Each group consisted of an equal number of male and female animals. Urine samples (in the first part of the project) and blood plasma (in the second part of the project) were subjected to NMR spectroscopy and multivariate statistical modeling. In total, two parts of the project form the first systematic description of the metabolomic response to opisthorchiasis in an animal model using two easily accessible biological fluids: urine and blood plasma.

In the first part of the project [33] urine samples were collected every two weeks for several months. The analysis showed that the most noticeable trend (30% of all deviations) in the data was related to gender differences; the body’s reaction develops depending on gender at the early stage of infection. Twenty-four metabolites associated with the observed effects were selected and several hypotheses were provided to search for more specific metabolomic markers of Opisthorchiidae infection [33].

In the second part of the project [34] blood plasma samples were collected one day before infection, and then every two weeks until 22 weeks after infection. The study showed that the plasma metabolomic response to Opisthorchis infection unfolds according to the same scenario as in urine, reaching its peak at the 4th week and stabilizing after 10 weeks after infection. However, in contrast to the reaction described in urine the observed metabolomic response in plasma is less specific for gender. The main directions of the metabolic response to infection in blood plasma are short-term depletion of essential amino acids and an increase in the concentration of lipoproteins and cholesterol [34].

## 8. Potato Plant Metabolomics

The metabolic processes occurring in potato plants are studied in the joint work of the group of scientists from St. Petersburg—Vavilov Research Institute of Plant Industry, Saint Petersburg State University and the Komarov Botanical Institute.

According to the FAO (Food and Agricultural Organization of the United Nation), potatoes represent the fourth most important food crop in terms of production after rice, wheat, corn and the first among tubers and root crops. The importance of potatoes is difficult to overestimate; it is a valuable source of carbohydrates, antioxidants, and vitamins. In recent years, a large number of studies have focused on the study of the potato metabolome for deciphering the mechanisms responsible for the productivity and accumulation of compounds that determine the taste and nutritional qualities, while maintaining the quality of tubers, plant resistance, etc. [35]. Complex studies of metabolic diversity using modern chromatographic approaches and high-precision NMR spectroscopy and mass spectrometric detection of individual compounds have revealed the specificity of metabolomic spectra both at the cellular and organism levels under the influence of both internal and external stimuli [36]. Metabolomic approaches are used for phenotyping available lines and varieties of potatoes, assessing the resistance of potato plants to environmental factors, and detecting changes in tubers during long-term storage [36]. Metabolomic profiling is widely used to study the differences between genetically modified forms of potato and non-transformed parent plants. In the future, studies of potato metabolome will be able to complement traditional and molecular genetic approaches to breeding to create new lines and varieties that carry valuable features [35].

Scientists from St. Petersburg have shown that the metabolome of potatoes, *Solanum phureja Juz. & Bukasov*, at the flowering stage totals 234 compounds, of which 117 are identified [37]. The most represented group among them contains sugars and their derivatives, which is consistent with the intensive carbohydrate metabolism of potato tissues and organs. Young leaves and developing reproductive organs are characterized by a wide range of organic and amino acids, nitrogen-containing compounds and lipids, as well as secondary metabolism compounds, which may indicate the intensity of metabolic processes and the formation of protective mechanisms. The depletion of the metabolomic profile in aging leaves is consistent with the idea of weakening the synthetic processes in them and starting the outflow of metabolites into forming the attracting organs of the potato. The specificity of metabolomic profiles corresponding to the age and physiological status of potato organs or tissues was revealed [37].

As mentioned above, ordinary potato, *Solanum tuberosum *L., is the fourth most important agricultural crop in the world. Until recently, vegetative propagation by tubers was the main method of potato cultivation. The shift in interest to sexual reproduction of potatoes by true botanical seeds is associated with the emergence of a new strategy for hybrid seed selection, the successful use of which for many types of crops was supported by male sterility. The metabolomic study was focused on the detection of differences in the profiles of anthers at the mature pollen stage from the male fertile and male sterile *Solanum tuberosum* L. genotypes [38]. The use of gas chromatography in combination with mass spectrometry revealed metabolomic profiles for 192 compounds. Further analysis of data with several libraries fully identified 75 metabolites; a similar amount was determined up to the class level. Metabolomic profiles of the anthers of fertile genotypes differ significantly from sterile ones by the accumulation of carbohydrates, while anthers of sterile genotypes contain more amino acids. Compared to male fertile plants, male sterile genotypes have undeveloped pollen grains; that is, a smaller grain size, a thicker exine, “permanent tetrads” that failed to disintegrate into microspores, and the absence of pollen apertures, which may be associated with a disorder of the metabolism of carbohydrates and fatty acids [38].

## 9. Current Trends in Metabolomics

Over the past decade, metabolomics has closed an important gap in the post-genomic research [4,66]. Determining the flow of information from the genome to the transcriptome, proteome and, finally, the metabolome allows, for the first time, to comprehensively describe living systems. However, to create models and datasets for accurate modeling, metabolic pathway reconstructions and highly efficient empirical metabolomic research technologies are essential.

A large number of scientists around the world have focused on metabolomics and the research methods and application of metabolomics have been optimized [67]. The Metabolomics Society was created in 2004 as a result of the growing interest in metabolomics. It was established to bring together, under one roof, scientists from the study areas representing metabolite target analysis, metabolic profiling, including metabolic fingerprinting or footprinting, metabolic flux analysis, biochemical modeling, and related bioinformatics fields. In 2007, the Metabolomics Standards Initiative (MSI) formed a general consensus on minimum reporting standards for metabolomics experiments [67]. Until now, all over the world, including Russia, these standards are used to monitor and develop protocols for standardization of the metabolic studies workflow and general analysis approaches.

With continuous optimization and improvement of high-tech research methods, metabolomics has become a wide area for the use of “-omics” technologies for medical purposes, the results of which are promising for future implementation [12,68,69]. With the support of the National Cancer Institute (NCI), Food and Drug Administration (FDA), and others, the Institute of Medicine (IOM) has been called upon to define the criteria and procedures for assessing the validity of tests created for the clinic. The IOM appointed the Committee on the Review of Omics-Based Tests for Predicting Patient Outcomes in Clinical Trials composed of the experts with wide range of knowledge and experience, including experts in “-omics” technologies. The committee presented the results of its activities in the book “Evolution of Translational Omics: Lessons Learned and the Path Forward”, published in 2012 [70]. As a result of the committee’s work, the criteria for preparing of “-omics” tests for clinical trials in humans have been created. The creation of “-omics” tests in accordance with these criteria, in particular, the writing of new protocols for them, reflects the modern understanding of the way to implement these tests into clinical practice. The studies of scientists from Russia on the development of laboratory tests for Parkinson’s disease, impaired glucose tolerance, lung cancer and prostate cancer made a great contribution to this direction [17,18,19,20,21,51,52].

It is worth noting another one initiative recently formed by The Metabolomics Society due to the fact that the study of metabolism at the global or “-omics” level is a rapidly growing field that has the potential to have a profound impact upon medical practice [71]. However, today clinicians utilize only a very small part of the information contained in the metabolome, as they routinely measure only a narrow set of blood chemistry analytes to assess health and disease states [71]. It is expected that “the narrow range of chemical analyses in current use by the medical community today will be replaced in the future by analyses that reveal a far more comprehensive metabolic signature. This signature is expected to describe global biochemical aberrations that reflect patterns of variance in states of wellness, more accurately describe specific diseases and their progression, and greatly aid in differential diagnosis” [71]. The work of scientists from Russia dedicated to metabolomic blood analysis, human digital imaging, and label-free data standardization for clinical metabolomics is aimed at supporting this initiative [23,24,25].

## 10. Conclusions

Being the youngest “-omics” science, metabolomics managed to establish itself in various fields of research, and the achievements of Russian metabolomics made tangible contributions to this. It can be confidently stated that metabolic studies in Russia are widely distributed and cover various biological objects and are carried out following the highest international standards. At the same time, Russian metabolomics has its priority research objects and original scientific works, which gives it a distinctive and interesting character, not allowing it to become lost in the general intensive flow of metabolomics research around the world. The role of IBMC as a growth point for metabolomic studies in Russia and the main place for medical metabolomics should be noted. The work carried out by the staff of this institute is the main pool of scientific publications of Russian metabolomics today.

## Figures and Tables

**Table 1:** The use of metabolomics in various fields of science in Russia.

Metabolomic Studies	Scientific Institutions on the Basis of Which Metabolomic Studies Are Conducted	References
METABOLOMIC STUDIES OF HUMAN BLOOD		
Metabolomic diagnostic signatures	Laboratory of mass spectrometry metabolomic diagnostics, IBMC (Moscow) together with:	[17] Prostate cancer
	− Endocrinology Research Center (Moscow)	[18] Impaired glucose tolerance
	− National Medical Research Center of Oncology (Moscow)	[19,20] Lung cancer
	− Institute of Developmental Biology (Moscow) and Kazan State Medical University (Kazan)	[21] Parkinson’s disease
	− Scientific Research Institute of Nutrition (Moscow)	[22] Obesity
Metabolomic profiling of human blood	Laboratory of mass spectrometry metabolomic diagnostics, IBMC (Moscow)	[23,24,25]
Therapeutic drug monitoring	Laboratory of mass spectrometry metabolomic diagnostics, IBMC (Moscow)	[26,27]
METABOLOMICS OF AGING PROCESSES		
	Laboratory of mass spectrometry metabolomic diagnostics, IBMC (Moscow)	[28,29]
METABOLOMICS OF PATHOLOGICAL PROCESSES DEVELOPMENT		
Cataract pathogenesis	Laboratory of proteomics and metabolomics, International Tomography Center, Siberian Branch of RAS (Novosibirsk)	[30,31,32]
The metabolic basis of the host’s response to helminth infection	Laboratory of clinical metabolomics, Tomsk State University (Tomsk) together with Central Research Laboratory, Siberian State Medical University (Tomsk)	[33,34]
PLANT METABOLOMICS		
Metabolic processes occurring in potato plants	Saint Petersburg State University (Saint Petersburg) together with Vavilov Research Institute of Plant Industry (Saint Petersburg) and Komarov Botanical Institute (Saint Petersburg)	[35,36,37,38]
